# Role of Flex-Dose Delivery Program in Patients Affected by HCC: Advantages in Management of Tare in Our Experience

**DOI:** 10.3390/jcm13082188

**Published:** 2024-04-10

**Authors:** Andrea Paladini, Marco Spinetta, Roberta Matheoud, Andrea D’Alessio, Miriana Sassone, Riccardo Di Fiore, Carolina Coda, Serena Carriero, Pierpaolo Biondetti, Domenico Laganà, Roberto Minici, Vittorio Semeraro, Gian Mauro Sacchetti, Gianpaolo Carrafiello, Giuseppe Guzzardi

**Affiliations:** 1 Department of Interventional Radiology, Santissima Annunziata Hospital, 74121 Taranto, Italy; 2 Radiology Department, University Hospital “Maggiore della Carità”, 28100 Novara, Italy; marcospinetta90@gmail.com (M.S.); miri.sassone@gmail.com (M.S.); richidifiore@alice.it (R.D.F.); carolinacoda94@gmail.com (C.C.); 3 Medical Physics Department, University Hospital “Maggiore della Carità”, 28100 Novara, Italy; roberta.matheoud@maggioreosp.novara.it (R.M.);; 4 UOC Radiology, Fondazione IRCCS Cà Granda, Maggiore Hospital, 20122 Milan, Italy; serena.carriero@policlinico.mi.it (S.C.);; 5 Radiology Unit, Dulbecco University Hospital, 88100 Catanzaro, Italy; donlaga@gmail.com (D.L.); miniciroberto@gmail.com (R.M.); 6 SSD Interventional Radiology, S.S. Annunziata Hospital, 74121 Taranto, Italy; vittoriosemeraro@hotmail.it; 7 Nuclear Medicine Department, University Hospital Maggiore della Carità, 28100 Novara, Italy; gianmauro.sacchetti@maggioreosp.novara.it; 8 Operative Unit of Radiology, Fondazione IRCCS Cà Granda Ospedale Maggiore Policlinico, 20122 Milan, Italy; gianpaolo.carrafiello@unimi.it; 9 Unit of Interventional Radiology, Department of Radiology, Ospedale Maggiore della Carità, Corso Giuseppe Mazzini 18, 28100 Novara, Italy; giuseppe.guzzardi@maggioreosp.novara.it

**Keywords:** HCC, BCLC, TARE, SIRT, flex-dose, target-therapy

## Abstract

**Background**: Introduced in the latest BCLC 2022, endovascular trans-arterial radioembolization (TARE) has an important role in the treatment of unresectable hepatocellular carcinoma (HCC) as a “bridge” or “downstaging” of disease. The evolution of TARE technology allows a more flexible and personalized target treatment, based on the anatomy and vascular characteristics of each HCC. The flex-dose delivery program is part of this perspective, which allows us to adjust the dose and its radio-embolizing power in relation to the size and type of cancer and to split the therapeutic dose of Y90 in different injections (split-bolus). **Methods**: From January 2020 to January 2022, we enrolled 19 patients affected by unresectable HCC and candidates for TARE treatment. Thirteen patients completed the treatment following the flex-dose delivery program. Response to treatment was assessed using the mRECIST criteria with CT performed 6 and 9 months after treatment. Two patients did not complete the radiological follow-up and were not included in this retrospective study. The final cohort of this study counts eleven patients. **Results**: According to mRECIST criteria, six months of follow-up were reported: five cases of complete response (CR, 45.4% of cases), four cases of partial response (PR, 36.4%), and two cases of progression disease (PD, 18.2%). Nine months follow-up reported five cases of complete response (CR, 45.4%), two cases of partial response (PR, 18.2%), and four cases of progression disease (PD, 36.4%). No intra and post-operative complications were described. The average absorbed doses to the hepatic lesion and to the healthy liver tissue were 319 Gy (range 133–447 Gy) and 9.5 Gy (range 2–19 Gy), respectively. **Conclusions**: The flex-dose delivery program represents a therapeutic protocol capable of “saving” portions of healthy liver parenchyma by designing a “custom-made” treatment for the patient.

## 1. Introduction

Primary liver cancer is the sixth tumor in terms of worldwide incidence and the second in terms of mortality. Hepatocellular carcinoma (HCC) represents the most common primary liver malignancy (80% of cases) [1]. Etiology is very different, but HBV and HCV are the main risk factors in Western countries nowadays. However, in the last 10 years, an increase in NAFLD (Not Alcoholic Fatty Liver Disease) and NASH (Not Alcoholic Steatohepatitis) as well as in alcohol abuse has been reported in patients affected by HCC. The prognosis depends on clinical, laboratory, and radiologic parameters, but HCC is the second leading cause of cancer death after lung cancer in men with a five-year survival of 18% [2].

Despite being gold standard therapy, surgical resection and liver transplantation (OLT) are possible in less than 10% of patients affected by HCC [3].

Therefore, many patients who are not eligible for surgery need different kinds of treatments in order to deal with this pathology. Loco-regional treatments have been developed in the last three decades with the goal of obtaining tumor cytoreduction for downstaging cancer and/or achieving a “bridge effect” for surgical treatment [4].

The choice of locoregional treatment is not easy and depends on accurate patient selection.

Barcelona Clinical Liver Classification (BCLC) is an important tool to classify patients based on clinical, laboratory analysis, and radiological criteria. Nowadays, BCLC 2022 is the most important therapeutic roadmap for patients affected by HCC.

An important update in BCLC criteria has been introduced in 2022 [5]. From the interventional radiologist’s point of view, the most important variation is the introduction of trans-arterial radio-embolization (TARE) as a treatment option in patients affected by Very Early (Stage 0) and Early Stage (Stage A) HCC whenever surgery or percutaneous treatment is not feasible or in case of their failure [5].

Radioembolization is defined as the injection of micron-sized embolic particles loaded with a radioisotope by the use of percutaneous transarterial techniques in order to deliver high focal doses of radiation to cancers.

Radioembolization is delivered using either yttrium-90 (90Y) resin microspheres (SIR-Spheres; Sirtex Medical Limited, Sydney, Australia) and 90Y-glass microspheres (Therasphere; BTG International Canada Inc., Kanata, ON, Canada) that have different physical characteristics, as described in the literature [6].

In the last few years, holmium-166 (166Ho) microspheres became commercially available, but due to the different management their use is not widespread and the respective dosimetry is yet to be extensively studied [7].

A different way of developing TARE treatment starts from the concept that this complex therapy should be tailored specifically to the patient’s characteristics and each case of HCC. Consequently, the new protagonist of this treatment is not the radiopharmaceutical or its delivery, but the dosimetry.

The importance of personalized dosimetry to make TARE safer and more effective has been demonstrated in recent clinical studies and international guidelines [7,8].

These papers outline the need for a personalized approach for 90Y-microsphere activity prescription in treatment optimization to deliver the highest dose to the tumor while limiting the dose to the non-tumoral liver. Post-treatment dosimetry is also recommended to ultimately quantify the dose-response relationship and hence the success of the treatment.

The flex-dose delivery program is a peculiar planning of precalibration that allows the splitting of 90Y resin microsphere activity as needed, and this characteristic provides flexibility for changes in the treatment plan and allows customization of the dose and number of particles based on the tumor characteristics [9].

Following the latest recommendation for selective ablative treatments, it is strongly recommended to consider a higher specific activity, hence delivering a lower number of microspheres with a higher activity per microsphere [10].

According to this program, SIR-Spheres are available in 4-day, 3-day, 2-day, or 1-day precalibrated vials. Each vial contains 44.5 million microspheres on average and on the day of calibration, the activity reached about 3.6 GBq. This leads to a huge variety of delivery options allowing tailored activity for patient-specific needs and making it possible to adapt the activity until the very last minute.

Moreover, thanks to the partition of the dose in the malignant vascular afferents, it is possible to obtain a greater concentration of the dose in the target lesion, reducing the dose to the healthy liver tissue.

In this paper, we report our experience with the Flex Dose Sir Spheres program, highlighting its efficacy through a dose RATIO (target dose/healthy liver tissue dose) and evaluating the efficacy of TARE treatment using the mRECIST criteria (6 and 9 months after the procedure).

## 2. Materials and Methods

### 2.1. Patients

A monocentric retrospective study was performed at the University of Eastern Piedmont (Novara, Italy) in the last two years, from January 2020 to January 2022.

One hundred and eighty-two patients affected by hepatic lesions were enrolled and evaluated by a multidisciplinary board (interventional radiologist, radiologist, hepatic surgeon, nuclear medicine physician, radiotherapist, and oncologist). During the multidisciplinary board, each patient was globally evaluated (performance status, anesthetic risk, presence of gastric varices, and/or portal hypertension) to choose the best therapeutic management.

One hundred and thirty-three patients were affected by unilobar or bilobar HCC (Stage 0: 8 patients, Stage A: 52 patients, Stage B: 43 patients, Stage C: 19 patients, Stage D: 11 patients following BCLC 2020). The remaining forty-nine patients of the cohort were affected by cholangiocarcinoma (9 patients), FNH (Focal Nodular Hyperplasia, 6 patients), atypical metastasis (25 patients), and large adenoma (9 patients). Finally, the multidisciplinary board selected nineteen patients affected by HCC Stage B for radioembolization treatment, (14 men; 5 women) with a mean age of 75.6 years (range: 56–85 years old).

Inclusion criteria for radioembolization were: >18 years old and ≥6 months life expectancy; BCLC Stages A, B, or C; ECOG performance score of 0 or 1; ≥33% of liver volume disease free; no extrahepatic disease or contraindications to angiography; no prior liver resection and/or transplant; absence of ascites; bilirubin level < 2 mg/dL, platelets count > 50,000, international normalized ratio (INR) < 1.5.

All patients were affected by cirrhosis of different etiologies: 5/19 (26.3%) exotoxic cirrhosis (alcohol, toxins, and drugs), 7/19 (36.8%) cirrhosis related to HCV infection, 7/19 (36.8%) affected by HCV associated with exotoxic etiology. In 89.5% of cases (17 patients), an increase in alfa-feto protein was detected.

The HCC diagnosis and its staging were obtained through a CT scan with contrast and a liver MRI scan, useful for a global study of each patient and a better description of TNM.

In 2 patients (10.5% of cases) only, a biopsy was needed due to an atypical radiological pattern of the lesion.

In our experience, the goal of radioembolization treatment was to achieve the downstaging of disease. In any case, TARE treatment was chosen as a bridge therapy to liver transplantation.

In 31% of cases (6 patients), it was not possible to perform the therapeutic treatment after simulation. A total of 5 patients, indeed, reported the presence of significative extra-hepatic shunts (3 cases with a pulmonary shunt > 20%, 1 case of pancreatic shunt, and 1 case of small-bowel shunt). Another patient died two days after the simulation from a cerebral hemorrhage (Table 1).

### 2.2. Treatment Simulation

A preliminary angiographic study was performed to simulate the radiopharmaceutical distribution in the liver finalized to study the vascular anatomy of the patient and to evaluate the optimal activity of the therapeutic agent to administer to the patient. Despite the literature reporting several mismatches between the simulation with 99mTc-MAA and the therapy distribution of 90Y-microspheres [11], the use of MAA is strongly encouraged for dosimetric evaluation and therapy optimization [11].

About 150 MBq of technetium-99m macro aggregated albumin (99mTc-MAA) was administered through the hepatic artery feeding the tumor.

The eventual presence of gastrointestinal shunting was investigated according to EANM guidelines [12] by acquiring a whole-body scan (LEHR collimator, 12 cm/min speed) on the Symbia-EVO gamma camera (Siemens Healthineers, Erlangen, Germany). Prior to the MAA scan, perchlorate was administered to prevent gastric uptake of free 99mTc, aiding in the interpretation of any potential gastric shunt. The lung shunt fraction (LSF) was calculated as the percent ratio between the geometric average of the total counts in a region of interest (ROI) drawn on the lung region (Counts_lung_) and the sum of the total counts in a ROI drawn on the liver (Counts_liver_) and the total lung counts:

LSF = (Counts_lung_)/(Counts_lung_ + Counts_liver_) % (1)

In our study, a preventive embolization was necessary in 6 patients (31% of cases) in order to obtain an “arterial flow redistribution” toward the hepatic target lesion and to prevent possible irradiation of non-tumoral tissues.

Subsequently, SPECT images of the liver/abdomen region of the patient were acquired on the same gamma camera (LEHR collimator, 3° angular sampling, 128 × 128 matrix size, 20 s/angular samplings) and reconstructed by use of the Flash3D iterative algorithm with scatter correction (3 subsets × 10 iterations, 8 mm Full Width at Half Maximum Gaussian filter). Finally, a low dose CT acquisition (X-ray tube current modulation with a Dose Right Index of 9/16, 50/80 reference mAs, 120 kV tube voltage, 0.83 spiral pitch factor, 40 mm collimation) was performed on the Ingenuity-TF 64 system (Philips Healthcare, Cleveland, OH, USA). To match the co-registration between SPECT and CT, three zeolites (radio-opaque markers) were placed on the patient’s skin at the sternum and bilaterally at the last rib, before SPECT and CT acquisitions. The procedure of zeolite activation has already been described in detail [13].

The dose evaluation was performed according to the three-compartment MIRD formalism [14], according to which the administered activity is distributed evenly within the normal and the tumor compartments. Moreover, the tumor compartment receives a higher activity concentration proportional to the tumor-to-liver ratio (TLR), which has been determined by using region-of-interest analysis of tumor and normal liver compartments on SPECT images.

The segmentation of the tumor, whole liver, and perfused liver on the co-registered SPECT-CT image dataset allowed the evaluation of the correspondent volume and mass (liver density = 1.03 g/mL).

According to [14], the fractional uptake in the normal liver and in the tumor were evaluated with the following formulas:

(2) $${fractional\text{\_}uptake}_{liver}=\left(1-LFS\right)\cdot [\frac{m_{liver}}{\left(m_{tumor}\cdot TLR\right)+m_{liver}}]$$

(3) $${fractional\text{\_}uptake}_{tumor}=\left(1-LFS\right)\cdot [\frac{TLR\cdot m_{tumor}}{(m_{tumor}\cdot TLR)+m_{liver}}]$$

where

*m_liver_*: mass of the normal liver;
*m_tumor_*: mass of the tumor.

These were used to calculate the maximum administrable 90Y-microsphere activities matching the dose constraints to lungs and normal liver, i.e., 20 and 40 Gy, respectively:

(4) $$A_{admin}\left(lungs\text{\_}20\mathrm{Gy}\right)=\frac{20[\mathrm{Gy}]\cdot m_{lungs}[\mathrm{kg}]}{49.7[\mathrm{Gy}\cdot \frac{\mathrm{kg}}{\mathrm{GBq}}]\cdot LSF}$$

(5) $$A_{admin}\left(liver\text{\_}40\mathrm{Gy}\right)=\frac{40[\mathrm{Gy}]\cdot m_{liver}[\mathrm{kg}]}{49.7[\mathrm{Gy}\cdot \frac{\mathrm{kg}}{\mathrm{GBq}}]\cdot {fractional\text{\_}uptake}_{liver}}$$

A lung mass of 1 kg based on the anthropomorphic phantom design applied in MIRD modelling was used [14]. In case of re-treatment, the cumulative lung mean dose is 50 Gy.

Finally, the activity of Y90microsphere to administer for a planned dose to the tumor (*D_tumor_*) was evaluated with the following formula:

(6) $$A_{admin}[\mathrm{GBq}]=\frac{D_{tumor}\left[\mathrm{Gy}\right]\cdot m_{tumor}\left[\mathrm{kg}\right]}{49.7\left[\mathrm{Gy}\cdot \frac{\mathrm{kg}}{\mathrm{GBq}}\right]\cdot fractiona{l\text{\_}uptake}_{tumor}}$$

The planned mean dose to the tumor must be in the range of 150–400 Gy. The lower limit of 150 Gy was based on the estimated mean dose required to achieve a complete response (CR) in previous studies [15,16]. In the unlikely event that the planned mean dose to the total tumor compartment cannot exceed 150 Gy, the patient is considered a screening failure.

### 2.3. TARE Procedure

In our hospital, radio-embolization treatment was performed using Y90 resin spheres (Sir Spheres^®^, Sirtex Medical Limited, Sydney, Australia). Each procedure was performed using the “Flex Dose” program with 3-day precalibrated vials, which allows the interventional radiologist to get the flexibility to deliver the same activity with a variable sphere concentration according to the characteristics of the tumor.

The microcatheter tip during Y90 resin spheres delivery was placed at the same anatomical position from which 99mTc MAA had been administered and the infusion of Y90 resin spheres was controlled under fluoroscopy to evaluate the distribution of contrast media in order to avoid pathological refluxes or non-target embolization.

In the 13 procedures performed, we did not report any technical failure or any intra- or postoperative complications achieving technical success in all patients treated (100%).

### 2.4. After Radioembolization

Post-therapeutic imaging was acquired soon after the treatment on the Ingenuity-TF PET/CT system (Philips Healthcare, Cleveland, OH, USA) by setting two-bed positions on the liver region with a total scan duration of 20 min. PET images were reconstructed with the OSEM iterative algorithm (99 equivalent iterations, TOF kernel of 14.1 cm, 4 mm Full Width at Half Maximum Gaussian filter, relaxation parameter set to 1.0) on a 144 × 144 frame (4 mm isotropic voxel).

Similarly to the simulation phase, segmentation of the tumor, perfused, and whole liver was performed on the PET/CT imaging dataset to evaluate the corresponding average doses for treatment verification.

### 2.5. Patient Follow-Up

A radiological follow-up was performed to evaluate the radiological outcome of the treatment. The radiological follow-up consisted of a CT scan of the abdomen with i.v. contrast at 6 and 9 months after radioembolization. The follow-up CT scans were performed on a 256-slice CT (PHILIPS Brilliance ICT, Philips Medical Systems, Cleveland, OH, USA) and all images were reconstructed with a slice thickness of 2.5 mm.

All patients enrolled have been evaluated by a radiologist with more than 10 years of experience in abdominal CT scans. The radiological outcome was assessed using modified Response Evaluation Criteria in Solid Tumors (mRECIST) [14], which was evaluated 6 and 9 months after therapy.

The radiological follow-up was performed on 11 patients. Two patients treated did not join the follow-up for reasons unrelated to liver disease (Table 1).

### 2.6. Endpoint

The primary endpoint of this study is to highlight the safety and the efficiency of the Flex Dose Sir Spheres delivery program assessing the ratio between the average dose to the tumor and the healthy liver tissue and evaluating CT scan response to the treatment using mRECIST criteria at 6 and 9 months after the procedure.

### 2.7. Statistics Analysis

This is a retrospective report on a small cohort of patients treated in a single hospital. Consequently, descriptive statistics were conducted considering the whole sample in order to evaluate the average absorbed dose, the ratio of the tumor/non-tumor average dose, and mRecist criteria at 6 and 9 months.

## 3. Results

Between January 2020 and January 2022, we analyzed one hundred and thirty-three patients affected by HCC, of whom nineteen were eligible for TARE treatment. The study participants (14 men and 5 women) had a mean age of 75.6 years (range: 56–85 years).

A total of 5 patients (26.3%) did not complete TARE treatment due to the presence of extrahepatic shunts after simulation treatment with Tc-MAA. A few days after simulation treatment, 1 patient (5.2%) died from a spontaneous cerebral hemorrhage. A total of 2 patients (10.5%), on the other hand, did not complete the radiological follow-up. The hepatic lesion weight in patients treated, calculated through CT volume 3D-analysis, was 0.265 kg (range: 1.090–0.041 kg).

The average absorbed doses planned for the hepatic lesion and for the healthy liver tissue were 319 Gy (range 133–447 Gy) and 9.5 Gy (range 2–19 Gy), respectively (Table 2).

It is worth noting that the ratio between the average dose to the tumor and the healthy liver tissue is 50.14 on average (range 10–200), outlining the high precision of the endovascular procedure in patients enrolled.

All thirteen procedures did not have any technical or equipment failures. No major complications were encountered during the post-procedure period; there was no radio induced liver failure and/or radio-induced pneumonia or cholecystitis. Moreover, there was no iatrogenic complication during the arterial puncture or during the catheterization.

Following the scientific protocol accepted by the Ethics Committee and subscribed to by all the patients, a radiological follow-up with abdomen-CT (6 and 9 months after the procedure) was performed to evaluate the outcome through mRECIST criteria (Table 3).

A total of 6 months after treatment, the radiological follow-up of the treated patients reported 5 cases of complete response (CR, 45.4%), 4 cases of partial response (PR, 36.4%), and 2 cases of progression disease (PD, 18.2%).

A total of 9 months after treatment, the radiological follow-up of the treated patients reported 5 cases of complete response (CR, 45.4%), 2 cases of partial response (PR, 18.2%), and 4 cases of progression disease (PD, 36.4%).

In two cases, a PR to therapy evolved to PD due to the presence of residual disease in the target lesion which increased in the last radiological follow-up.

In two cases, PD was detectable in the first radiological check due to the presence of extra-hepatic disease and the presence of another pathological tissue near the target lesion.

Patients with extrahepatic PD started systemic chemotherapy, while patients with locoregional PD underwent chemoembolization. In two patients with PR at six months and PD at nine months with new hepatic nodules, locoregional thermal ablation treatment was opted for.

Two patients did not complete the follow-up table and therefore were not enrolled in the study (Table 3).

## 4. Discussion

The development of new oncological therapies together with the update of BCLC 2022 [17] depicted a new role for radioembolization in the management of HCC.

Especially in cases where the treatment target is downstaging, we believe that it is essential to obtain a “segmental” therapy that follows the concept of surgical segmentectomy [18]. In these cases, the goal of treatment should be to concentrate the radiopharmaceutical in the tumor, trying to obtain dose-sparing in the adjacent healthy tissue. 

Unfortunately, as each HCC has a unique morphology and vascular anatomy, TARE treatments and dose delivery can be limited by anatomical complexity [19,20,21]. Consequently, a new “philosophy” in the delivery of the dose has been developed.

In this paper, we report the high effectiveness of the “Flex Dose program” which allows the tailoring of a custom-made treatment for each patient and the delivery of a high radioactivity concentration in the target lesion (following the main concept of radioembolization, which is a kind of endovascular brachytherapy), preserving the healthy liver tissue.

In our experience, we were able not only to concentrate a high dose on the pathological lesion (319 Gy on average reported; range between 133 and 560 Gy) but also to “spare” the adjacent healthy liver parenchyma with an average ratio (absorbed dose in the target lesion/absorbed dose in the healthy liver) of 51 (range between 200 and 11). In every patient treated, therefore, the dose delivered to the tumor was far greater than the adjacent tissue. Flex-dose protocol, therefore, has allowed a precise and targeted treatment to preserve healthy tissue and liver function.

The paper of Levillain et al. [10] showed that the absorbed dose necessary to obtain a “radiant segmentectomy” and oncological downstaging treatment must be >150 Gy. In the present study, the dosimetric results show that the treatment was selective in all the patients treated in whom a radiant segmentectomy was obtained.

In the SARAH trial (using the BSA method), a post hoc 99mTc-analysis of the delivered dose based on MAA SPECT/CT showed that overall survival and disease control were significantly better with a tumor-absorbed dose > 100 Gy [15]. The probabilities of disease control at 6 months were 72% (95% CI 46–89%) and 81% (95% CI 58–93%) with tumor-absorbed doses of 100 Gy and 120 Gy, respectively [15,22].

However, these results do not only represent a simple exposition of procedural data but also present an important clinical implication for the management of the patient and his pathology.

In the loco-regional liver treatment with Y90 microspheres, indeed, there is increasing evidence of a correlation between the absorbed doses delivered and local lesion response and overall survival.

Lee et al. [16] evaluated the response of 42 patients who underwent TARE treatment using resin microspheres. The median delivered dose reported was 50.8 Gy. According to RECIST criteria, the 6-month disease control rate was 94% of cases.

Ho et al. [22] estimated the correlation between tumor-absorbed doses and responses in a group of 71 patients affected by HCC. Tumor doses were estimated by the partition model. The median absorbed dose at the first treatment was 225 Gy (range: 38–748 Gy). A total of 37% of the patients reported a partial response at absorbed doses > 225 Gy, in comparison with 10% at absorbed doses < 225 Gy.

Flamen et al. [23], on the other hand, demonstrated that the mean absorbed dose to the healthy liver parenchyma without toxicity was 39 Gy (32–48 Gy).

Strigari et al. [24] analyzed the outcome of 73 patients affected by HCC: with an average dose of 110 Gy to the tumor, complete or partial response was observed in 74 and 55% of patients according to the EASL and RECIST criteria, respectively.

According to mRECIST criteria, patients enrolled in our study showed a complete or partial response to therapy in 63.6% of cases in the 9-month follow-up. This value is very close to what the literature and other authors have reported in the last few years [18,20,23,25].

In the study of D’Arienzo et al. [25], the mean absorbed dose to the hepatic lesion was 139 Gy, but two areas were distinguished: a hot margin receiving an average absorbed dose of 287 Gy (range: 100–700 Gy) and a cold area with a necrotic core receiving an average absorbed dose of 70 Gy (with a large proportion of tissue receiving <50 Gy). At the FDG-PET control 6 months after treatment, complete remission was observed in highly irradiated areas, while progression of disease was observed in the scar cell irradiated area. It demonstrates a deep correlation between absorbed dose and radiological lesion response.

Thanks to the flex-dose delivery program, the interventional radiologist can perform not only a very effective treatment thanks to the high absorbed dose delivered in the lesion but also to obtain a significant healthy liver tissue sparing [26].

Starting from the concept that patients and HCC are different from each other, the flex-dose delivery program is the answer to different pathological anatomies. TARE treatment, indeed, starts being “tailor-made” on patient-specific anatomy and HCC pathological patterns [26].

Moreover, it permits different injection sites through the splitting of the absorbed dose delivered and—consequently—a better coverage of the lesion. In selected patients, this results in the capability of treating two different lesions in a single procedure or a single lesion with two different arterial feeders.

The flex-dose program permits us not only to “adapt” the radiant activity but also to check the delivery during the same angiographic procedure due to its visibility. Therefore, the TARE treatment could be stopped and restarted during the same procedure, and the catheter position can be easily modified.

The main limitations of this study are the small number of patients enrolled and the impossibility of making a comparison with TARE treatment with glass spheres due to the lack of experience with this type of therapeutic option.

Despite the aforementioned limits, we maintain that the concept of flexibility before and during the treatment is very important due to the different situations that the interventional radiologist could deal with.

## 5. Conclusions

The flex-dose delivery program represents a therapeutic protocol capable of sparing portions of healthy liver parenchyma by designing a “custom-made” treatment for the patient.

Personalized activity prescription, based on dosimetry and multidisciplinary management for optimization of safety and efficacy, is recommended when conducting TARE with 90Y resin microspheres.

Some dose-effect correlations are still unpredictable, but a strong correlation between absorbed dose delivered-radiological and clinical response, allows us to predict toxicity, radiological response, and patient survival. Due to the heterogeneity of each hepatic lesion, individualized dosimetry treatment is the new frontier of target therapy. A “flexible” radio-embolization treatment is necessary to improve the management of patients affected by HCC. Multidisciplinary management together with the flex-dose delivery program represents a therapeutic protocol able to preserve healthy liver parenchyma from radiant therapy, in the new concept of “custom-made” treatment for the patient.

## Figures and Tables

**Table 1:** Clinical characteristics of patients.

Patients	Gender	Age (Years)	HCC Etiology	HCC Characteristics	ALBI Score	BCLC	ECOG	Follow-Up
1	Female	67	exotoxic	unilobar	−2.42 Grade 2	B	0	Yes
2	Female	72	HCV	unilobar	−2.36 Grade 2	B	0	Procedure not performed
3	Male	78	HCV + exotoxic	bilobar	−2.42 Grade 2	B	0	Yes
4	Female	67	HCV	bilobar	−2.50 Grade 2	B	1	Yes
5	Male	69	exotoxic	unilobar	−2.69 Grade 1	B	0	Yes
6	Male	71	HCV	unilobar	−2.56 Grade 2	B	0	Yes
7	Male	65	exotoxic	unilobar	−2.95 Grade 1	B	1	Yes
8	Male	78	HCV + exotoxic	unilobar	−2.48 Grade 2	B	0	
9	Male	56	exotoxic	unilobar	−2.45 Grade 2	B	1	Yes
10	Male	70	HCV	unilobar	−1.91 Grade 2	B	0	Procedure not performed
11	Male	77	exotoxic	unilobar	−2.87 Grade 1	B	0	Yes
12	Female	72	HCV + exotoxic	unilobar	−2.50 Grade 2	B	0	Yes
13	Female	74	HCV + exotoxic	bilobar	−3.28 Grade 1	B	1	Procedure not performed
14	Male	59	HCV + exotoxic	unilobar	−3.59 Grade 1	B	0	Not completed
15	Male	66	exotoxic	unilobar	−2.78 Grade 1	B	0	Procedure not performed
16	Male	85	HCV + exotoxic	bilobar	−2.70 Grade 1	B	1	Yes
17	Male	76	HCV + exotoxic	unilobar	−2.57 Grade 2	B	0	Not completed
18	Male	74	HCV	bilobar	−2.96 Grade 1	B	1	Procedure not performed
19	Male	64	HCV	unilobar	−3.04 Grade 1	B	0	Procedure not performed

**Table 2:** Procedure data in patients enrolled.

Patients	Lung Shunt	90Y Activity MBq	Absorbed Dose Tumor (Gy)	Absorbed Dose Healthy Liver	Lesion Weight (kg)	Ratio	Tumor Size-Maximum Diameter (cm)
1	2.1%	1890	306	14	0.218	22	5.5
2	3%, patient dead						
3	1.45%	1130	158	14	0.066	11	3.7
4	1.45%	810	560	14	0.083	40	4.1
5	1.8%	1780	289	6	0.271	48	6.3
6	2.9%	640	350	10	0.041	35	3.2
7	1%	1700	400	2	0.206	200	5.5
8	3.7%	3280	133	13	1.094	10	10.5
9	6.2%	1420	290	9	0.422	32	7.9
10	6.1%	2800	447	5	0.144	89	4.5
11	28%, not enrolled						
12	1.1%	1520	261	5	0.266	52	6.3
13	21% not enrolled						
14	2.3%	1120	380	5.2	0.119	76	4.2
15	0.6% but evidence of pancreatic shunt						
16	4.4%	2590	296	19	0.361	33	7.3
17	2.4%	1280	285	8	0.165	36	5
18	26% not enrolled						
19	1% but evidence of small bowel shunt						
Average			319	9.5	0.265 kg		
Median			296	9	0.206 kg		
IQR			95	8.8	0.152 kg		

**Table 3:** Radiological results in patients treated with TARE and flex-dose delivery program.

Patients	Mrecist 6 Months Follow-Up	mRECIST 9 Months Follow-Up	ALBI Score before TARE	ALBI Score Post TARE	Notes
1	CR	CR	−2.42 Grade 2	−1.54 Grade 2	
2			−2.36 Grade 2		
3	PR	PR	−2.42 Grade 2	−1.54 Grade 2	
4	PR	PR	−2.50 Grade 2	−2.33 Grade 2	
5	CR	CR	−2.69 Grade 1	−2.30 Grade 2	
6	CR	CR	−2.56 Grade 2	−2.96 Grade 1	
7	PD	PD	−2.95 Grade 1	−2.62 Grade 1	Extra-hepatic pathology progression
8	PR	PD	−2.48 Grade 2	−2.56 Grade 2	Evidence of other hepatic nodules during follow-up
9	CR	CR	−2.45 Grade 2	−1.95 Grade 2	
10	PD	PD	−1.91 Grade 2	−1.72 Grade 2	Evidence of an increasing tissue near hepatic lesion
11	-	-	−2.87 Grade 1		
12	PR	PD	−2.50 Grade 2	−1.75 Grade 2	Increasing residual tissue after TARE and evidence of new hepatic nodules
13	-	-	−3.28 Grade 1		
14	CR	-	−3.59 Grade 1	−2.30 Grade 2	Absence of a complete follow-up
15	-	-	−2.78 Grade 1		
16	CR	CR	−2.70 Grade 1		
17	PR	-	−2.57 Grade 2	−2.70 Grade 1	Absence of a complete follow-up
18	-	-	−2.96 Grade 1		
19	-	-	−3.04 Grade 1		

## Data Availability

Data are contained within the article.
